# Standard Methods for Dissection of *Varroa destructor* Females

**DOI:** 10.3390/insects13010037

**Published:** 2021-12-29

**Authors:** Vincent Piou, Caroline Vilarem, Carolin Rein, Lina Sprau, Angélique Vétillard

**Affiliations:** 1 Laboratoire Evolution et Diversité Biologique, UMR5174, CNRS-Université de Toulouse III-IRD, INU Jean-François Champollion, Université Paul Sabatier, 31077 Toulouse, France; vincent.piou@univ-tlse3.fr (V.P.); caroline.vilarem@m2i-group.fr (C.V.); 2 M2i Biocontrol—Entreprise SAS, 46214 Parnac, France; 3 Apicultural State Institute, University of Hohenheim, Erna-Hruschka-Weg 6, 70599 Stuttgart, Germany; carolin.rein@uni-hohenheim.de; 4 Department of Livestock Population Genomics, University of Hohenheim, Garbenstr. 17, 70599 Stuttgart, Germany; lina.sprau@uni-hohensheim.de

**Keywords:** *Varroa destructor*, dissection, guidelines, pictures, videos

## Abstract

**Simple Summary:**

*Varroa destructor* (Anderson and Trueman) is known as a major pest of *Apis mellifera* L. Research about this parasite is very active and dynamic, yet only a few studies focus on an organ in particular. These targeted works are very important as they can enhance our comprehension of the mite, as they did for its host. Dissection is unavoidable when trying to isolate a specific organ. This technical article aims to give illustrated guidelines to conduct female *V. destructor* dissection. Thanks to photos and videos, this work will constitute a helpful tool to anyone interested in *V. destructor* dissection, anatomy, or organ function.

**Abstract:**

*Varroa destructor* (Anderson and Trueman) is known as a major pest of *Apis mellifera* L, especially in the Northern Hemisphere where its effects can be deleterious. As an obligate parasite, this mite relies entirely on its host to reproduce and complete its cycle. Studies focusing on isolated organs are needed to better comprehend this organism. To conduct such targeted molecular or physiological studies, the dissection of *V. destructor* mites is crucial as it allows the extraction of specific organs. Here, we propose a technical article showing detailed steps of females *V. destructor* dissection, illustrated with pictures and videos. These illustrated guidelines will represent a helpful tool to go further in *V. destructor* research.

## 1. Introduction

*Varroa destructor* is considered the major pest of the Western honey bee *Apis mellifera* causing important stress throughout the world and even colony losses in the Northern Hemisphere [1,2]. Females of this parasite feed on body fluids of the bee at the larval, pupal, and adult stages, transmitting pathogenic viruses in the process [2,3]. Because it is such an important issue for honey bees and beekeeping in many countries, more and more research is being conducted on different aspects of the host–parasite relationship [4]. The reproductive physiology of the mite and the many viruses vectored by the parasite are particularly studied [5,6,7,8,9,10].

However, only a few have focused on the *V. destructor* anatomy or on an organ in particular, the reproductive organs being the main exception [11,12,13,14,15,16,17,18]. Dissection and extraction of specific organs, as it is often performed on honey bees, allows more subtle investigations of the host–parasite interactions [11,12,13,19,20,21,22,23,24]. The reproductive status and fertility of mites can indeed be precisely assessed by microscopy once the genitalia are extracted [14,16]. This also allowed the detailed description of the capacitation process of spermatozoa inside the females spermatheca [15]. Depending on the organ considered, the *V. destructor* genes involved in the ecdysteroid synthesis pathway, in the foraging behavior or coding for heme-binding proteins, were all shown to vary in their expression [12,23,24]. The organ to be isolated thus has to be carefully chosen depending on the aim of the study. In the case of viruses transmitted by the mite, the extraction of specific organs can also be interesting, especially now that some viral clones were shown to replicate within mites [8,25]. This lack of organ-focused studies is partly due to the size and morphology of the parasite that can make the dissection dissuasive. *Varroa destructor* is indeed a 1 mm long mite with a very flattened and sclerotized body [1,26]. The difficulty may be emphasized by the fact that no recent detailed and illustrated dissection protocol is, to our knowledge, available. Here, we will present, for the first time, pictorial methods of *V. destructor* dissection adapted to the organ of interest, as it already exists for *A. mellifera* or for other species of Acari [20,27,28].

## 2. Material, Methods and Results of the Dissection Protocols

### 2.1. Morphology and Material

*Varroa destructor* is a 1.7 mm large, 1.1 mm long brown mite (Mesostigmata, Acari) [26,29,30]. Its flattened body is covered by articulate sclerotized plates, one large dorsal shield, and seven smaller ventral plates, which have to be removed to have access to the internal organs. To dissect such a little organism, the material, although limited, is of prime importance. Sharpened entomology pins (0.25 mm thick) and/or forceps (Dumont 5) are required to anatomize the ecto-parasite (Videos S1–S3). Sharpening stones specifically designed for Dumont tweezers (Fine science tool, Canada) are necessary to maintain the precision of the dissection tools. The dissection has to be performed under a stereomicroscope with an appropriate magnification (between ×10 and ×30). In this study, the stereomicroscope (Leica S8APO) was connected to a camera (Leica MC 190HD) and a screen (Iiyama, all three components from Leica Microsystems, Nanterre France) to take pictures and record videos. All the methods presented here were conducted according to the European ethics laws for scientific research currently in force (i.e., the Directive 2010/63/EU of the European Parliament and of the Council of 22 September 2010 on the protection of animals used for scientific purposes).

In this study, female *V. destructor* were sampled at any time of their cycle, from the start of the reproduction in brood cells to the dispersal phase on adults. However, to avoid the presence of eggs or embryos in photos and videos, most females were collected on adult bees during their dispersal phase or during the later stages of reproduction (i.e., on worker pupae from day 8 to 12 after the sealing of the cell). The first step of the dissection consists of freeze killing the female *V. destructor* in a −20 °C freezer for five minutes before fixing the mites on a microscope slide or on top of a Petri dish filled with ice. The latter can be very useful as low temperatures allow the conservation of organs. Several fixation methods are described in the literature using wax, glue, or double-sided tape [11,16,31,32]. In this guide, we used cyanoacrylate gel glue as we found liquid glue less practical to precisely fix the mite. Regardless of the method used, the steadiness of the mite is crucial to succeed. When using glue, a 10 to 20 min drying period is necessary. Depending on the organ that has to be isolated, the parasite can be fixed either on its ventral or on its dorsal shield. Nevertheless, we recommend dorsal dissection as it allows most organs to be extracted without damage.

### 2.2. Ventral Dissection for the Isolation of Reproductive Organs

**Figure 1:**
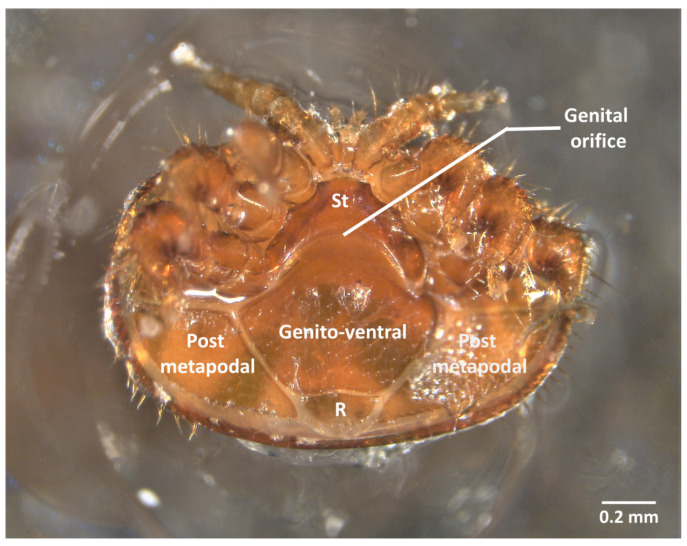
External view of a *V. destructor* female glued before ventral dissection (R = rectal scutum; St = sternal scutum).

If the acari is glued on its dorsal scutum, then it will be dissected ventrally (Video S1 and Figure 1). The majority of studies about the reproductive organs of *V. destructor* use ventral dissection [14,16,17]. Once the ecto-parasite is fixed dorsally and covered with 30 µL of phosphate buffered saline (PBS 1× Gibco™: KH_2_PO_4_ 1.06 mM, NaCl 155.2 mM, Na_2_HPO_4_-7H_2_O 2.9 mM) the dissection begins with the removal of the genito-ventral scutum. This step is carried out by softly introducing the pin or forceps in the genital opening and sliding under the scutum following the intersegmental membrane between the sternal and genital scutum (Video S1 and Figure 1). Slightly lifting the pin when it is under the scutum will limit the damage caused to the underlying organs. The genital scutum can then be seized with forceps and pulled to extract it along with the rectal scutum. Note that removing the rectal scutum will tear the rectum, which leads to the release of its content (Video S1). The sliding then continues laterally following the membrane between the anterior and posterior metapodal scuta. These posterior scuta are then seized and extracted following the same process (Figure 1 and Video S1). We recommend stopping the removal of scuta there, as the further removal of the anterior metapodal scuta or legs of the mite will most generally damage the digestive tract underneath and does not help genitalia extraction. Regardless of the mode of dissection, when the female is sampled during its reproduction, oocytes or embryos can occupy the genital tract of the female (Figure S1). Oocytes are located in the ovary and can reach a diameter of 300 µm after vitellogenesis [18]. According to Steiner et al. 1994 [18], the first egg/oocyte becomes an embryo as soon as the cleavage phase starts, around 30 h after cell capping. During the honey bee late prepupal stage, the first embryo migrates further in the genital tract and is often found in the oviduct right next to the genital opening (Figure S1a). Depending on the stage of the embryos, limbs can be more or less identifiable [18] (Figure S1b,c).

**Figure 2:**
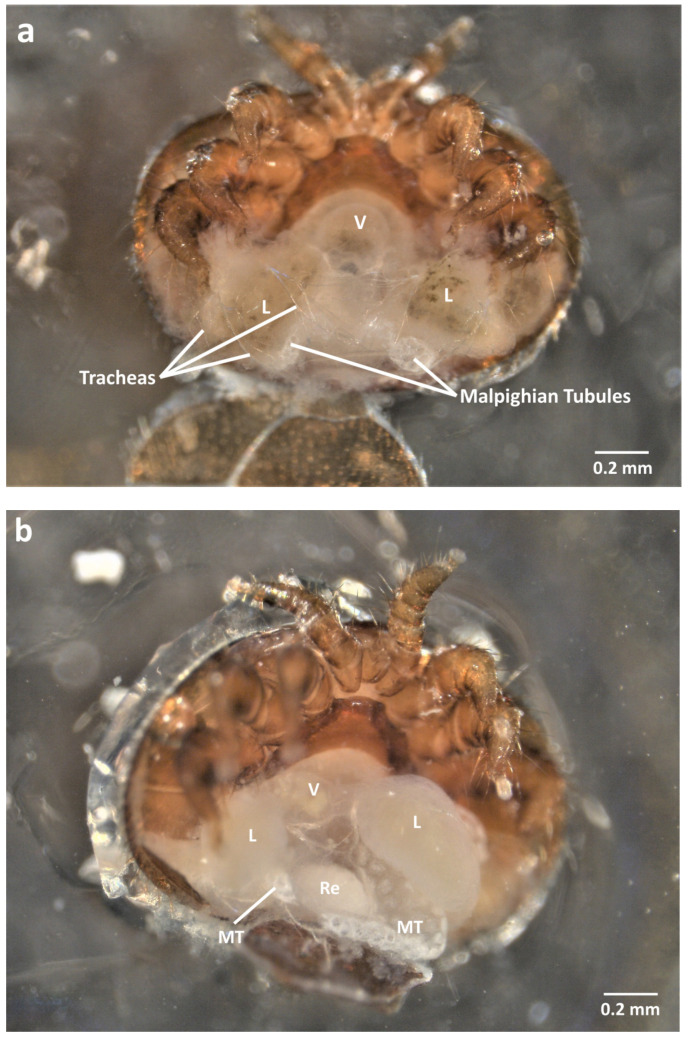

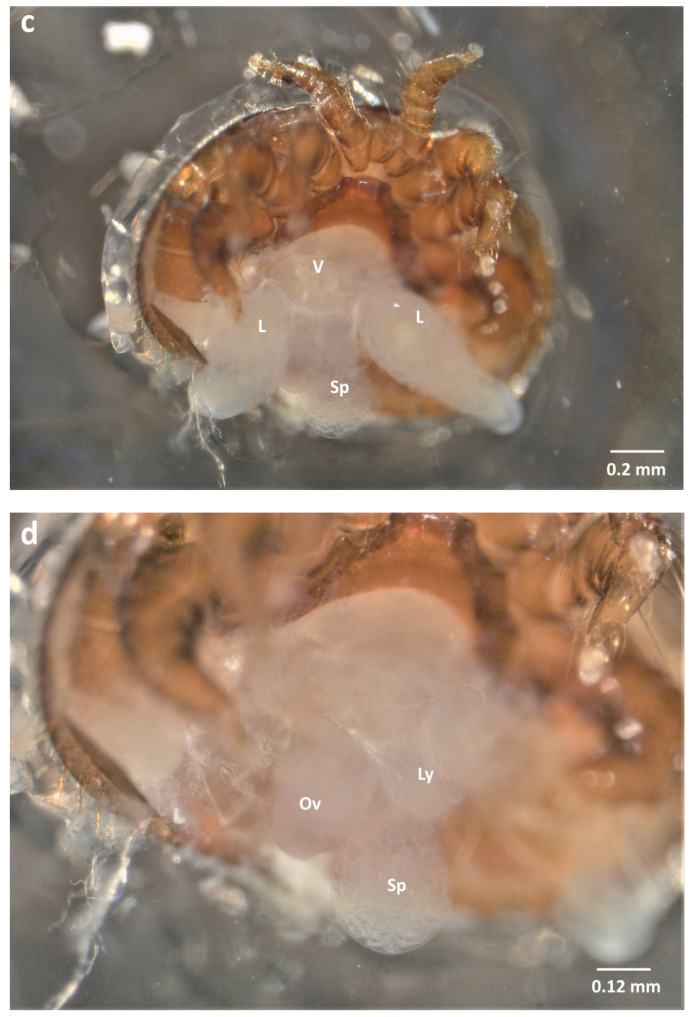

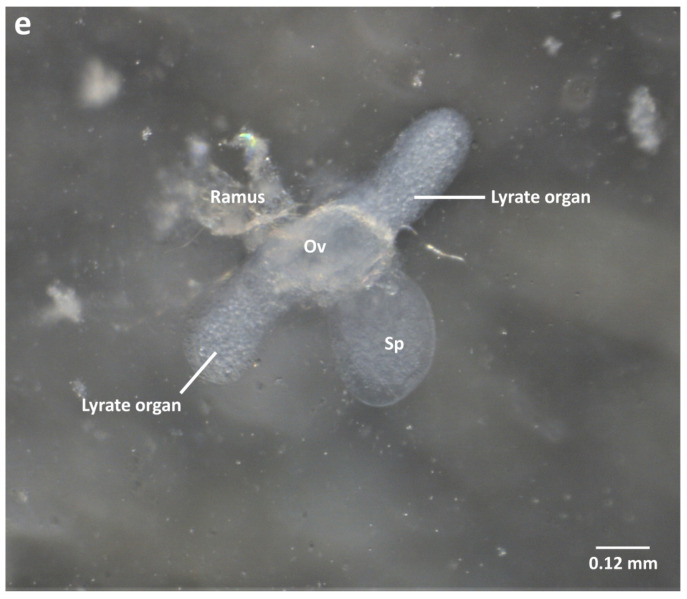
Pictures of the internal anatomy of reproductive *V. destructor* females (sampled on late-stage honey bee pupae) during ventral dissection (**a**) view after the genito-ventral scutum and the posterior metapodal scuta are removed, tracheas cover the Malpighian tubules and the caecal lobes (L), (**b**) after the layers of tracheas and fat bodies are removed, the caecal lobes (L) and Malpighian tubules (MT) are accessible (**c**) once the Malpighian tubules are extracted, the reproductive organs can be seen (**d**) after both posterior caecal lobes are extracted, the reproductive organs are now accessible (**e**) Extracted reproductive organs of a female *V. destructor* (L = caecal lobes; Ly = lyrate organs; Ov = ovary; MT = Malpighian tubules; Re = rectum; Sp = spermatheca; V = ventriculus).

Once the genito-ventral and posterior metapodal scuta are removed, the organs are discernible under a veil-like layer of tracheas and fat body tissues (Figure 2a and Video S1). The tracheas have to be carefully pulled out using pins or forceps. The digestive system then appears more clearly (Figure 2b and Video S1). Laterally, the two posterior caecal lobes, part of the mite’s digestive system, are visible as transparent yellowish masses while the excretory Malpighian tubules occupy a more central position and appear as white strings (Figure 2b). The Malpighian tubules can be swollen or narrow, depending on the physiological state of the mite, and guanine granules can sometimes be seen [33,34]. The Malpighian tubules are the first organs to be removed. Although breakable, slightly pulling on the tubules is sufficient to remove the whole organs. The posterior caecal lobes are then easily discernible and can be naturally enlarged and colored depending on the repletion of the acari (Figure 2c). By gently pulling each lobe, the removal of the posterior part of the digestive tract allows the isolation of the reproductive organs (Figure 2d,e).

Although transparent, the genitalia can be seen in a central position and are composed of the spherical spermatheca linked to the more ventral ovary. The lyrate organs form two lateral growths emerging from the ovary (Figure 2e). These organs are thought to have a nutritive role for the growing oocyte [18,35,36,37]. The fertilization of the oocyte is also thought to occur in the lyrate organs. Even if the two rami are hard to keep intact during dissection, they can sometimes be identified as two strings that end up joining in a spermatic duct attached to the spermatheca (Figure 2e and Figure S2). The rami are a sperm-access system that host spermatozoa after mating [37]. They were also shown to be transitory spermatozoa capacitation organs [14,15].

### 2.3. Dorsal Dissection

**Figure 3:**
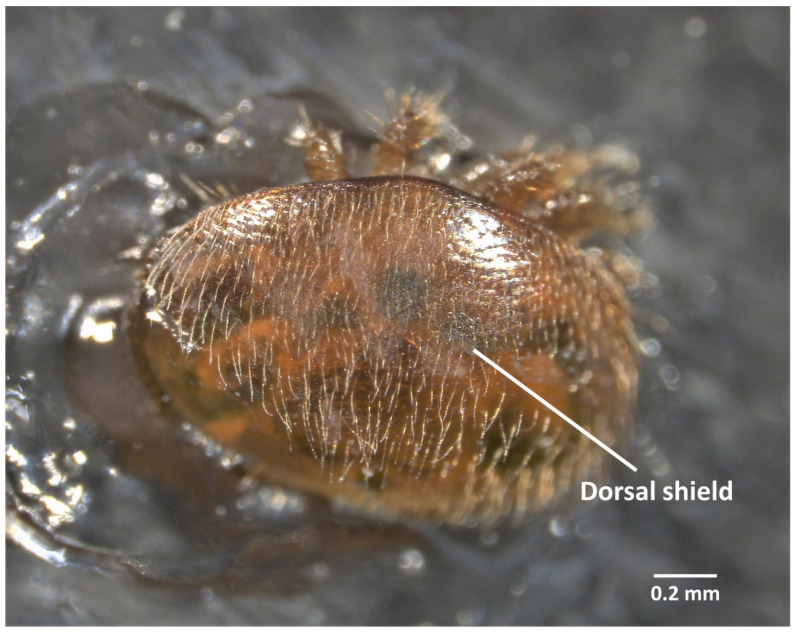
External dorsal view of a *V. destructor* female before dissection.

In this case, the mite is stuck ventrally and submerged in 30 µL of PBS (1×) and the dorsal scutum has to be removed to have access to the internal organs (Figure 3, Videos S2 and S3).

**Figure 4:**
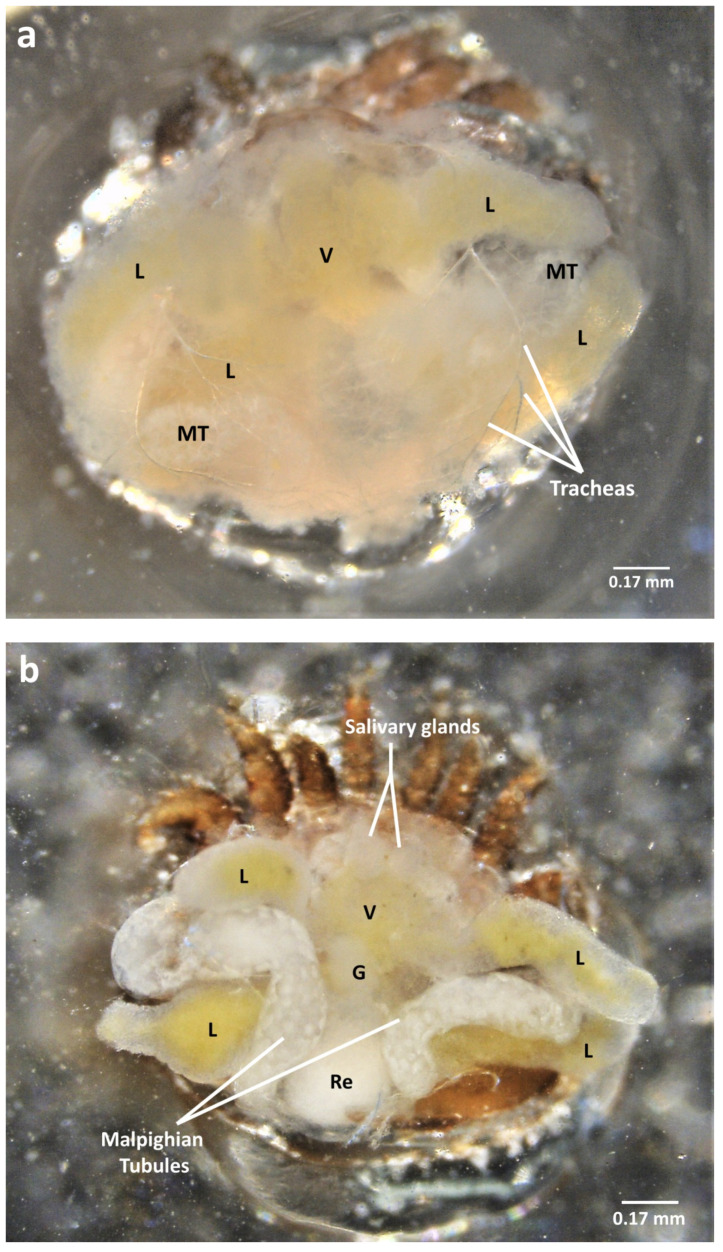

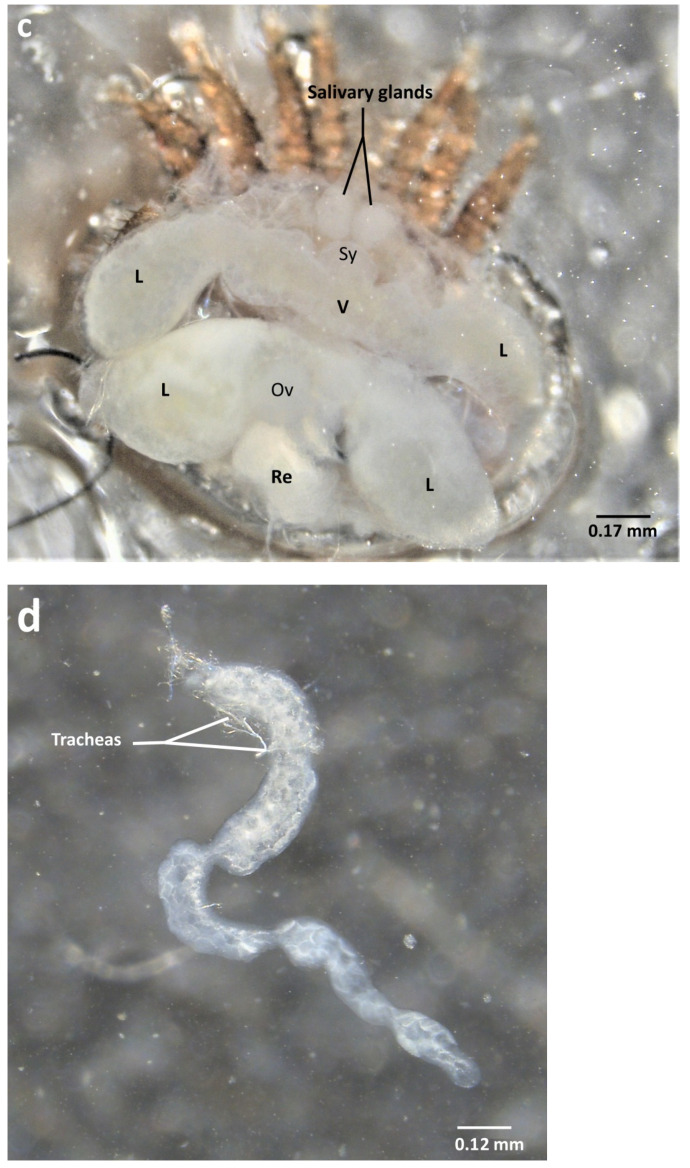

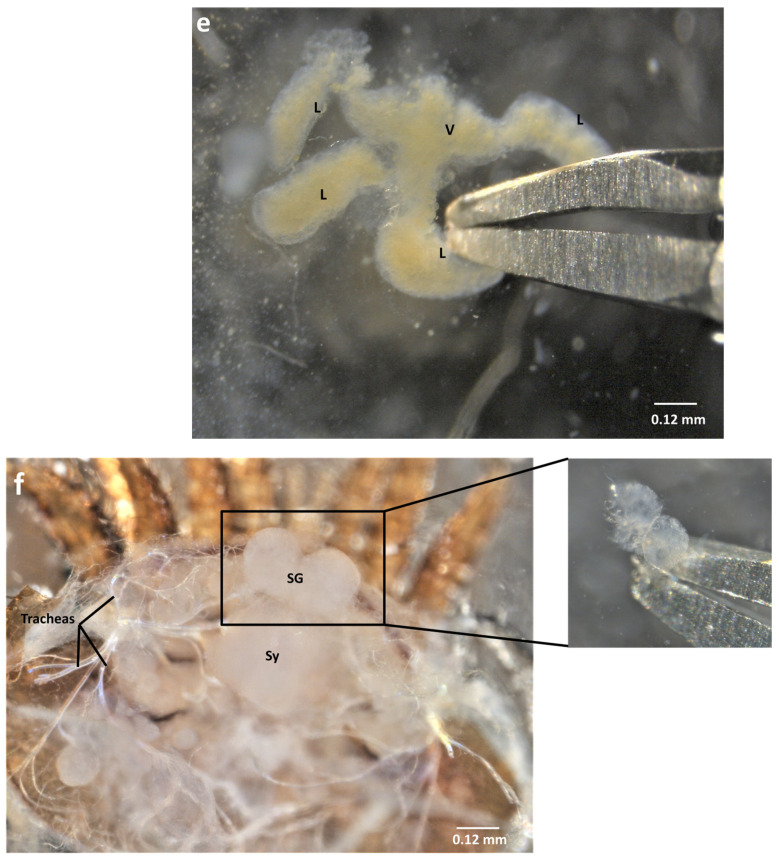
Pictures of the internal anatomy of dispersive or reproductive *V. destructor* females (sampled on adults or honey bee pupae, respectively) during dorsal dissection; (**a**) view after the dorsal shield is removed; (**b**) after the layer of tracheas and fat bodies are removed; (**c**) once the Malpighian tubules are extracted; (**d**) Malpighian tubule removed; (**e**) extracted digestive tract of the mite (without the rectum); (**f**) salivary glands observed within the mite’s body and once extracted. (L = caecal lobes; MT = Malpighian tubules; Ov = ovary; Re = rectum; SG = salivary glands; Sy = synganglion; V = ventriculus).

By softly sliding the tip of a sharp pin or forceps on the lateral, anterior, and/or posterior sides of the dorsal shield, one can carefully lift it (Videos S2 and S3). Again, the organs are visible but hidden under a layer of tracheas and fat body tissues which thickness depends on the physiological state of the mite (Figure 4a, Figure S3, and Video S3). Once removed, the digestive organs, along with the Malpighian tubules, are clearly identifiable (Figure 4b,c and Video S3). The Malpighian tubules consist of two long strings between the other organs, some parts being covered with tracheas and some thinner and more fragile. The tubules can still be carefully pulled out and their ends cut. This will inevitably lead to the loss of a part of the content but the organs can still be preserved and used for further analyses (Figure 4d). The digestive tract is now exposed. Anteriorly, it is composed of a short and thin esophagus that goes through the synganglion. The esophagus is followed by a central ventriculus from which four caecal lobes (two anterior and two posterior) emerge laterally. The ventriculus is also attached posteriorly to the rectum (Figure 4b,c). The genitalia, located above the junction between the ventriculus and the rectum, are also extractable dorsally (Figure 4b,c, Figures S2 and S3, Videos S2 and S3). To separate the digestive tract from the genitalia, the method is to clearly identify both systems and try to seize and softly pull the ventriculus (Figure 4e and Video S2). The digestive tract should come without harming the genitalia. The rectum will most often be sampled separately as it does not remain attached when the rest of the digestive tract is extracted. Anteriorly, two transparent white spheres are discernible in a central position, right above the gastropharyngeal pump and next to the synganglion. These are the salivary glands that can also be sampled for diverse analyses (Figure 4f and Video S2).

### 2.4. Organ Sampling and Preservation

After the dissection in PBS 1X, we suggest rinsing the removed organ in a clean PBS solution before transferring it into a sample tube. Depending on the objective of the study, different conservation solutions can be considered. For genes expression or transcriptomic projects, organs can be kept in RNA later provided sample tubes are then stocked at −80 °C [13,19]. Alternatively, the dissected organs can be directly transferred in extraction buffer or Trizol reagent and stored at −80 °C [11,12,28]. Thanks to the constant improvement in proteomic and metabolomic methods, storage in PBS 1X at −80 °C is now possible for such analyses although flash freezing in liquid nitrogen is recommended. Another option is to extract proteins on freshly collected organs and to store the vacuum-dried protein samples at −80 °C [38].

More direct downstream analyses consist of histological staining or observations under a light microscope or through sample preparation and electronic microscopy observations. Organs can again be transferred in a PBS solution before analyses. Brief storage at 4 °C is possible even though it is better to work on fresh tissues and organs for these types of analyses [14,15,16,17,18].

## 3. Discussion

In this article, we aspire to show the reality of females *V. destructor* dissection, without any staining, drawing, or artifice. The idea is to illustrate what any scientist cutting the mite open could observe. Several precise schemes are available in the literature [32,33,37,39]. These drawings represent an interesting starting point to learn *V. destructor* anatomy as they summarize in one picture the majority of internal organs. However, the mite is a 3D creature, thus under a stereomicroscope, it is impossible to see all organs at once with a clear focus. This is why the photos and videos presented here constitute a perfect complement to these schemes to comprehend *V. destructor* dissection. In this work, we focused on female adult stages of the mite since males and immature stages only occur briefly during the reproductive phase. The dissection of adult males or female deutonymphs, although possible, is more difficult due to the soft unsclerotized cuticle which requires a precise incision to be removed.

When studying mature females, we suggest using the dorsal dissection in the first place. Compared to ventral dissection, dorsal dissection is more straightforward as it requires the removal of only one scutum. The dorsal region of females *V. destructor* indeed has no anatomical orifice. As a result, the underlying organs are not altered by the dissection and the majority of internal organs can be extracted using dorsal dissection. More precisely, the Malpighian tubules, tracheas and fat bodies, digestive tract, genitalia, synganglion, and salivary glands were all extracted following the dorsal dissection steps presented here. The muscle layer is also discernible via the methods described here, even if the extraction of *V. destructor* legs is probably better to isolate muscular tissues. The heart cannot be collected using dissection but other methods to investigate *V. destructor* heart were already described [40]. Ventral dissection is more complex and we recommend it only for studying the reproductive organs, as it was used successfully in several research works [14,15,16,17].

The video presenting dissection methods of *V. destructor* females also gives an insight into the inter-individual variability. The aspect of caecal lobes and Malpighian tubules indeed depends on the physiological status of the mite. The tubules can appear as swollen tubes with clearly visible white guanine granules or as shrunk white strings [33,34]. The caecal lobes can also be more or less swollen and be yellow, white, or green depending on their nutritional status. It would be interesting to further investigate the molecular basis of the differential aspects taken by the caecal lobes and Malpighian tubules. These physiological differences might emerge, at least partially, from the stage of the mite’s development. Although the overall difficulty of the dissection is similar between the reproductive and dispersal stages, this variability can make organ identification more complex. During the prepupal stage, reproductive mites are swollen and have a high amount of fat bodies, which makes the removal of scuta easier. However, the presence of eggs or embryos occupying a large portion of the mite’s body can disturb the identification of organs. On the contrary, the dispersive mites have low fat reserves and carry no eggs in their genital tract. This makes the removal of scuta slightly more difficult but the identification of organs easier. The late reproductive mites, found on honey bee pupae between the brown-eyed and the dark pigmented body stage, can represent an interesting compromise to start learning mite dissection. In any case, practice is crucial to becoming familiar with *V. destructor* anatomy.

In conclusion, dissection and isolation of organs are important to improve our understanding of *V. destructor*. This can lead to a more subtle level of analysis in studies regarding reproduction [14,15], physiology or organ-specific gene expression [11,12,13,19]. Organs have indeed specific patterns of genes expression and can respond differently to a change of environment [23,24]. Working at the organ scale thus represents a complementary approach to studies at the individual or colony level. This first pictorial guide gives a precise overview of the dissection to help identify the different organs as they will appear in reality. It will undoubtedly represent a helpful tool that can be used by many researchers in their studies about the widespread honey bee parasite *V. destructor*.

## Data Availability

All relevant data (pictures and videos) is displayed in this article. The raw footages and photos are available upon request.

## Supplementary Materials

The following are available online at https://www.mdpi.com/article/10.3390/insects13010037/s1: Figure S1. (a) Embryo appearing during a ventral dissection of a reproductive female *V. destructor* sampled on a prepupal honey bee worker; (b) early stage of the embryo during the limb bud formation phase [18]; (c) late stage of another embryo after limb differentiation [18]; Figure S2. Extracted genitalia of females *Varroa destructor* sampled on adult bees after dorsal dissection (a) natural aspects after dissection; (b) after a 30 s Eosin bath (2%); Figure S3. Steps of the dorsal dissection after the mite ingested a blue FCF-dyed diet (following the methods from [41,42]). Video S1. Extraction of reproductive organs of females *Varroa destructor* through ventral dissection; Video S2. Dorsal dissection of a female *Varroa destructor*; Video S3. Video of females *Varroa destructor* dissection. The females used in the videos were sampled either at the end of their reproductive phase or on adult bees.
