# Supplementary material for: Standard Methods for Dissection of Varroa destructor Females
Source: Insects. 2021 Dec 29;13(1):37. doi: 10.3390/insects13010037 (PMC8781925; doi:10.3390/insects13010037)
Supplement: Supplementary file 1 [file insects-13-00037-s001.zip › insects-1507890-supplementary.pdf]

Online Supplementary Material

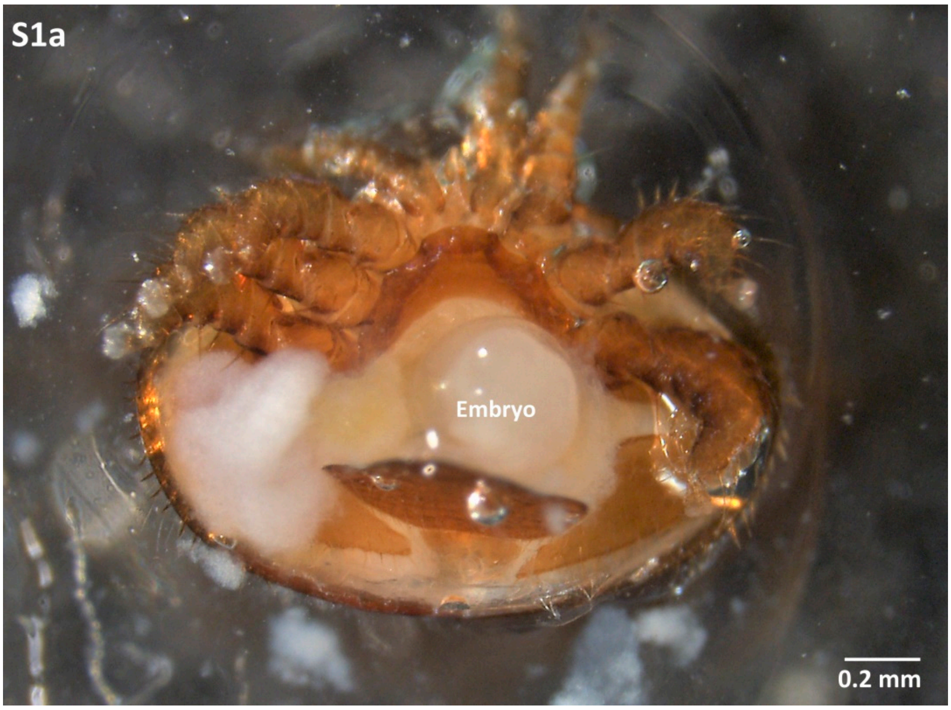

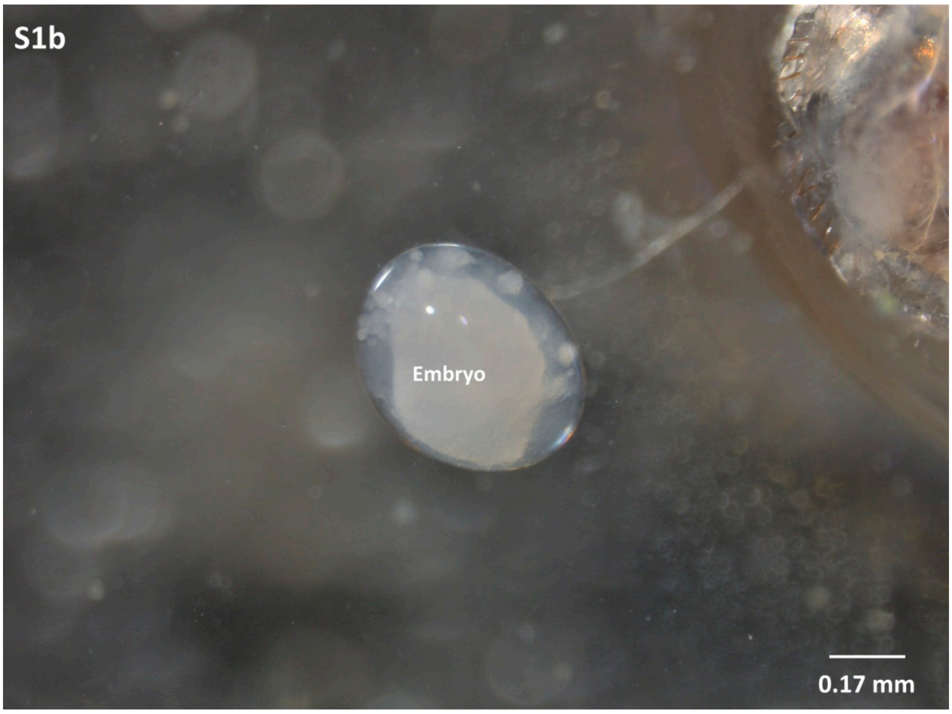

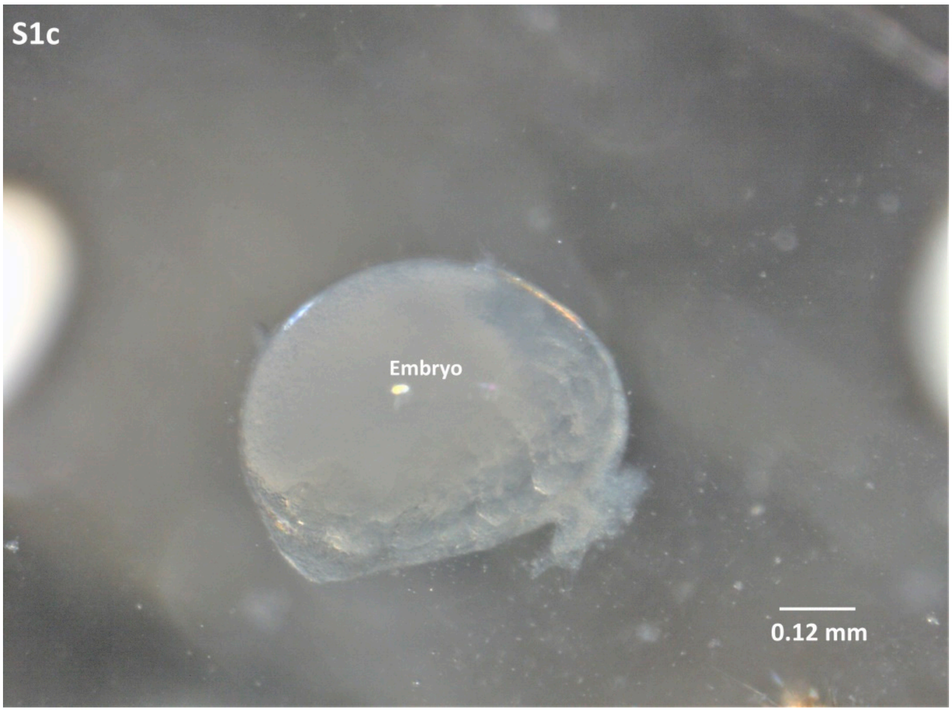

Figure S1. a) Embryo appearing during a ventral dissection of a reproductive female *V. destructor* sampled on a prepupal honey bee worker; b) early stage of the embryo during the limb bud formation phase [18] c) late stage of another embryo after limb differentiation [18]

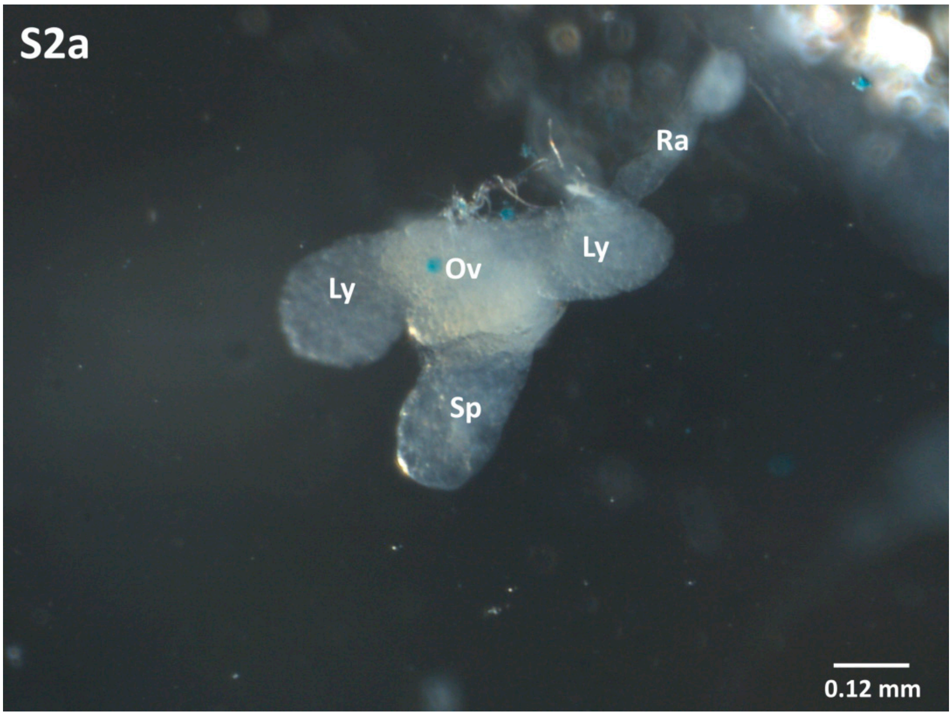

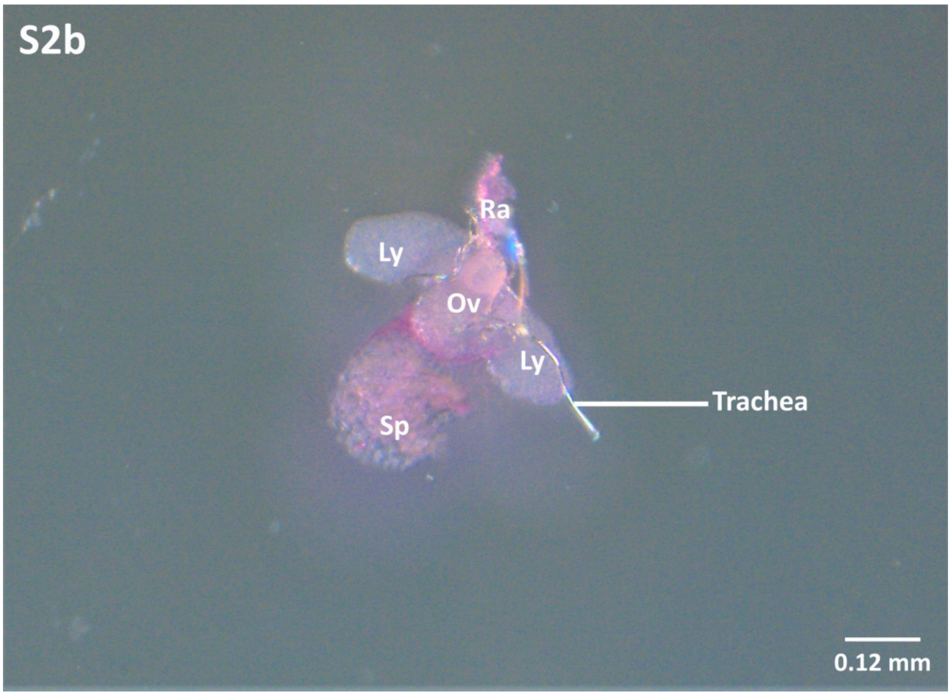

Figure S2. Extracted genitalia of females *Varroa destructor* sampled on adult bees after dorsal dissection a) natural aspects after dissection b) After a 30s Eosin bath (2%) (Ly = Lyrate organs; Ov = Ovary; Ra = Ramus; Sp = Spermatheca)

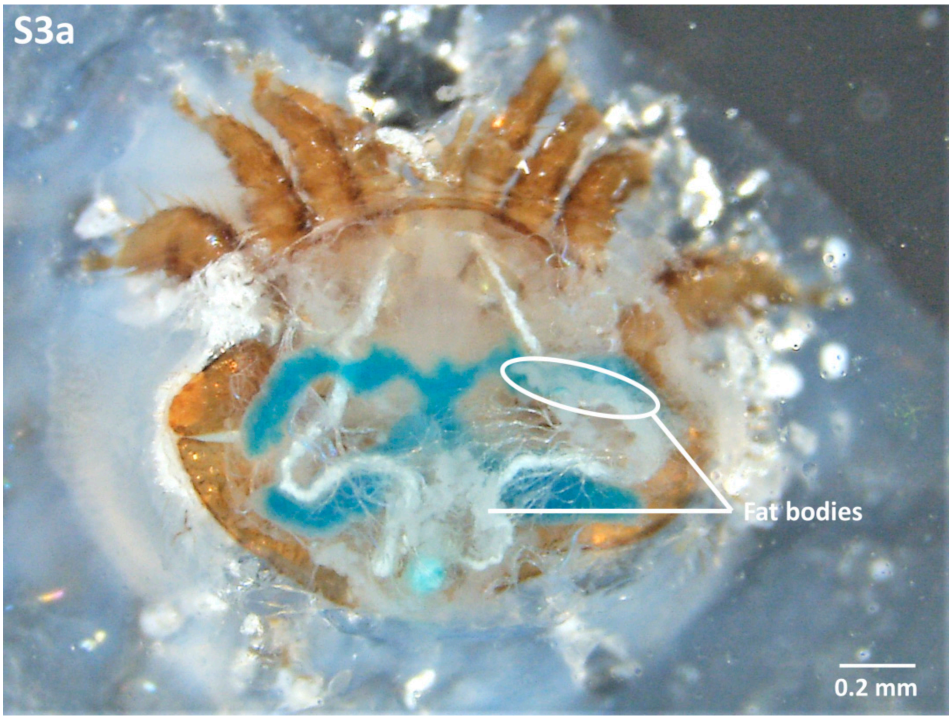

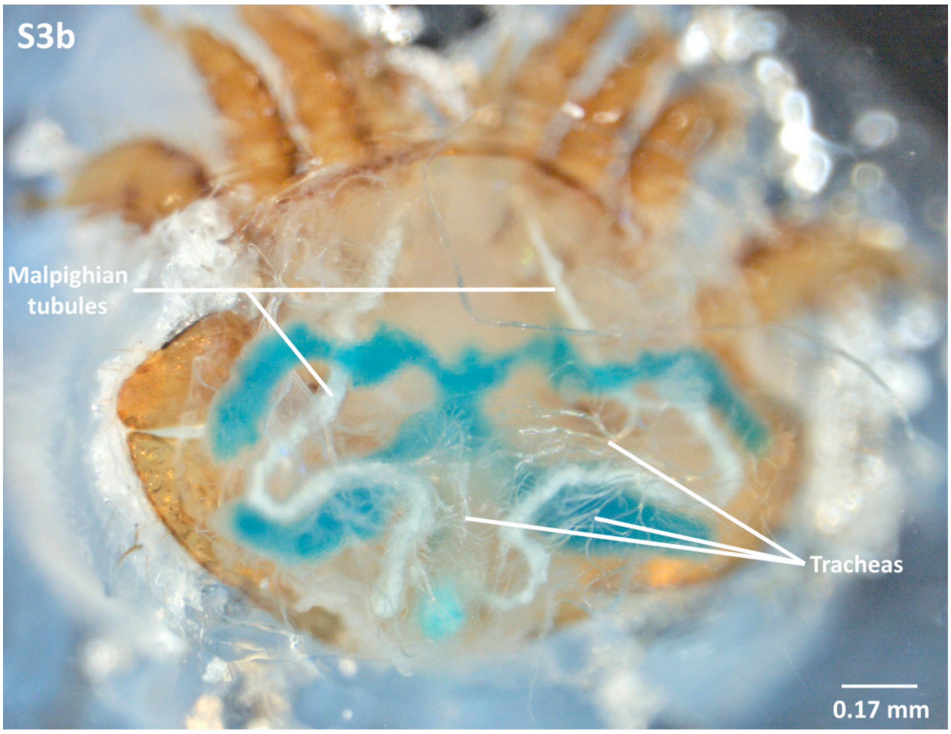
S3b
Malpighian tubules
Tracheas
0.17 mm

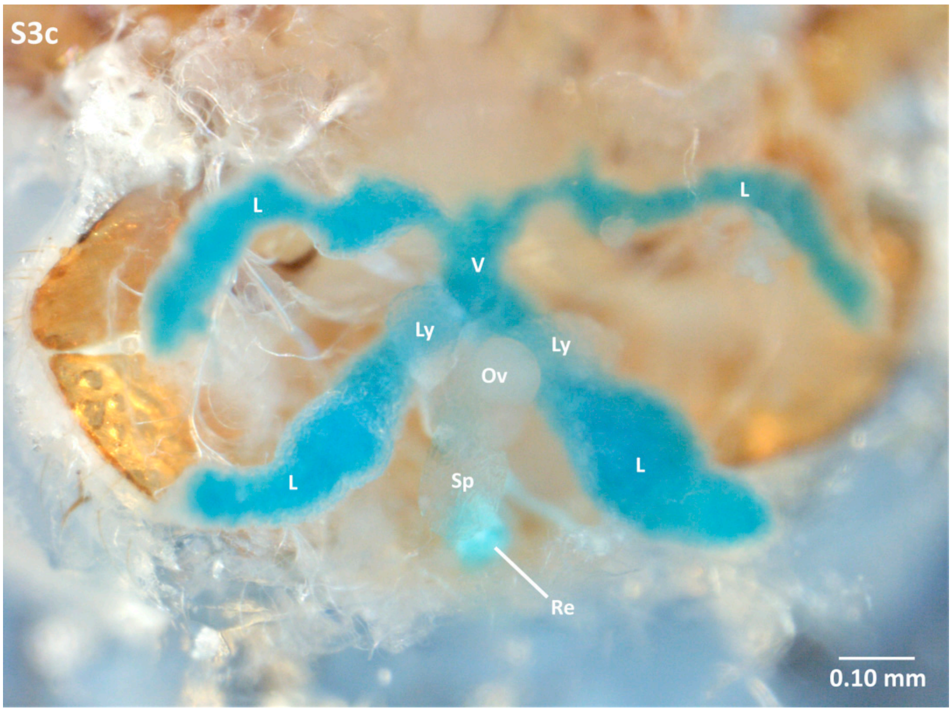
S3c
L
L
V
Ly
Ly
Ov
L
L
Sp
Re
0.10 mm

Figure S3. Steps of the dorsal dissection after the mite ingested a Blue FCF dyed diet (following the methods from [41,42]). a and b) Once the dorsal scutum is removed, fat bodies and tracheas cover the organs in the posterior region of the mite's body and have to be carefully extracted, c) The removal of this layer along with the Malpighian tubules allows the observation of the digestive tract (Blue) and of the reproductive organs (L = caecal lobes; Ly = Lyrate organs; Ov = Ovary; MT = Malpighian Tubules; Ra = Ramus; Re = Rectum; Sp = Spermatheca; Tr = Tracheas; V = Ventriculus)

Supplementary videos

https://zenodo.org/record/5725174#.YcuX02hKg2w

Video S1. Extraction of reproductive organs of females *Varroa destructor* through ventral dissection

Video S2. Dorsal dissection of a female *Varroa destructor*

Video S3. Video of females *Varroa destructor* dissection.

The females used in the videos were sampled either at the end of their reproductive phase or on adult bees
